# Declining Trend of Transapical Access for Transcatheter Aortic Valve Replacement in Patients with Aortic Stenosis

**DOI:** 10.1155/2022/5688026

**Published:** 2022-09-19

**Authors:** Sumit Sohal, Harsh Mehta, Krishna Kurpad, Sheetal Vasundara Mathai, Rajiv Tayal, Gautam K. Visveswaran, Najam Wasty, Sergio Waxman, Marc Cohen

**Affiliations:** ^1^Division of Cardiovascular Diseases, Department of Medicine, RWJ-BH Newark Beth Israel Medical Center, 201 Lyons Ave, Newark, NJ 07112, USA; ^2^Division of Cardiovascular Diseases, Department of Medicine, University of Kansas Medical Centre, 3901 Rainbow Boulevard, Kansas, KS 66160, USA; ^3^Department of Medicine, RWJ-BH Saint Barnabas Medical Center, 94 Old Short Hills Rd, Livingston, NJ 07039, USA; ^4^Department of Medicine, NYC Health+Hospitals/Jacobi Medical Center, 1400 Pelham Pkwy S, Bronx, NY 10461, USA; ^5^Division of Cardiovascular Diseases, Department of Medicine, Valley Health System, 1200 East Ridgewood Avenue West Wing, Suite 301, Ridgewood, NJ 07450, USA

## Abstract

**Introduction:**

The last decade has witnessed major evolution and shifts in the use of transcatheter aortic valve replacement (TAVR) for severe aortic stenosis (AS). Included among the shifts has been the advent of alternative access sites for TAVR. Consequently, transapical access (TA) has become significantly less common. This study analyzes in detail the trend of TA access for TAVR over the course of 7 years.

**Methods:**

The national inpatient sample database was reviewed from 2011–2017 and patients with AS were identified by using validated ICD 9-CM and ICD 10-CM codes. Patients who underwent TAVR through TA access were classified as TA-TAVR, and any procedure other than TA access was classified as non-TA-TAVR. We compared the yearly trends of TA-TAVR to those of non-TA-TAVR as the primary outcome.

**Results:**

A total of 3,693,231 patients were identified with a diagnosis of AS. 129,821 patients underwent TAVR, of which 10,158 (7.8%) underwent TA-TAVR and 119,663 (92.2%) underwent non-TA-TAVR. After peaking in 2013 at 27.7%, the volume of TA-TAVR declined to 1.92% in 2017 (*p* < 0.0001). Non-TA-TAVR started in 2013 at 72.2% and consistently increased to 98.1% in 2017. In-patient mortality decreased from a peak of 5.53% in 2014 to 3.18 in 2017 (*p*=0.6) in the TA-TAVR group and from a peak of 4.51% in 2013 to 1.24% in 2017 (*p*=0.0001) in the non-TA-TAVR group.

**Conclusion:**

This study highlights a steady decline in TA access for TAVR, higher inpatient mortality, increased length of stay, and higher costs compared to non-TA-TAVR.

## 1. Introduction

Over the last decade, the management of severe aortic stenosis (AS) has seen a dramatic shift with several trials demonstrating the efficacy and safety of transcatheter aortic valve replacement (TAVR) and its use has now progressed from high-risk patients to include intermediate and low-risk patients [1–3]. The shift has not only been in the type of procedure performed but also in how the procedure is performed. Initially, only two sites, i.e., transfemoral (TF) access and transapical (TA) access, were commonly used to perform TAVR, but in the last decade, several new access sites came into practice, including but not limited to transcarotid, transaortic, transaxillary, and transcaval access sites [4–6]. Studies also suggested higher postprocedural adverse events in TA-TAVR and higher survival rates in TF-TAVR, which lead to the decline in use of TA access for TAVR [7–9].

The combination of these factors has affected the use of TA-TAVR, but no large-scale study has been done to study its trend. In this study, we use the national inpatient sample (NIS) database to study these trends and compare the in-hospital mortality, length of stay, cost, and postprocedure complications of TA-TAVR against non-TA-TAVR. These comparative analyses of outcomes will help us identify the factors and better understand their effect on the procedural volumes of TAVR done through TA access.

## 2. Methods

### 2.1. Data Source

Data used in this study was derived from the national inpatient database (NID), which is a subset of the healthcare cost and utilization projects and is funded by the Agency for Healthcare Research and Quality. NIS contains data on more than seven million hospital stays each year from more than 1000 hospitals, which approximates a 20% stratified sample of US community hospitals. In 2017, 48 states participated in NIS, covering more than 97% of the US population and nearly 96% of discharges from US community hospitals [10].

### 2.2. Study Population

For our study, we used the NIS database from the years 2011 to 2017. We used International Classification of Diseases, ninth revision, clinical modification (ICD-9-CM), and ICD-10-CM to identify our study population. All hospitalized patients above the age of 18 with a diagnosis of aortic stenosis during their stay were included in our study. During our search for ICD-9-CM and ICD-10-CM, codes were found only for TA-TAVR and TF-TAVR. For this study, any procedure other than TA access was classified as non-TA access as several physicians, for coding purposes, use TF-TAVR codes when using alternate access (non-TF and non-TA) sites. Thereafter, we queried the selected patients from the NIS database till 2015 using an ICD-9-CM code of 35.06 for TA-TAVR and 35.05 for non-TA-TAVR. Similarly, after 2015, ICD-10-CM codes of 02RF37H, 02RF38H, 02RF3JH, and 02RF3KH were used for TA-TAVR and 02RF37Z, 02RF38Z, 02RF3JZ, and 02RF3KZ were used for non-TA-TAVR. Discharge weights provided by HCUP were used to generate national estimates. The study protocol was exempted from human subject research as it included publicly available, deidentified data.

### 2.3. Outcomes

The primary outcome of our study was to study the trend of TA-TAVR when compared to non-TA-TAVR on hospitalized AS patients during the study period, i.e., 2011 to 2017. The secondary outcomes studied were to compare in-hospital mortality, length of stay, cost, and rates of complications in both arms. The complications that were included were cardiac complications, major bleeding, vascular complications, sepsis (infectious complications), stroke or transient ischemic attack (TIA), acute kidney injury (AKI) requiring dialysis, and permanent pacemaker implantation. The ICD codes utilized for these complications have been provided in the supplementary file (Table S1).

### 2.4. Statistics

STATA 13 (StataCorp LLC, Texas) was used to analyze the NIS database. Differences between categorical variables were tested using the *χ*^2^ test, and differences between continuous variables were tested using the Student's *t*-test or Wilcoxon rank-sum test. Categorical data are presented as a frequency in percentage and continuous data are presented as mean ± SD. Multivariable regression was used to predict the relationship between the independent and dependent variables. A 2-sided *p* value of <0.05 was considered to be significant.

## 3. Results

A total of 3,693,231 patients were identified with a diagnosis of aortic stenosis. 129,821 patients underwent TAVR, out of which 10,158 (7.82%) underwent TA-TAVR and 119,663 (92.18%) underwent non-TA-TAVR. Patients who underwent TA-TAVR were younger (79.71 ± 0.23 years vs. 80.65 ± 0.06 years, *p*=0.001). The baseline characteristics and underlying comorbidities of these patients are outlined in Tables 1 and 2.

**Figure 1:**
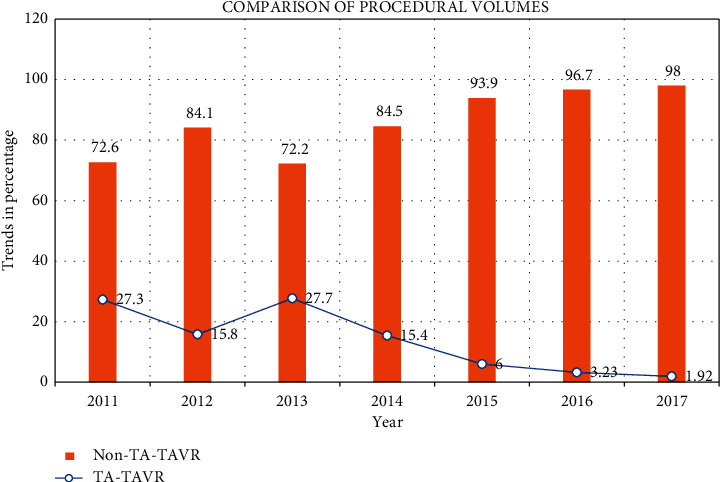
Comparison of procedural volumes from 2011–2017.

**Figure 2:**
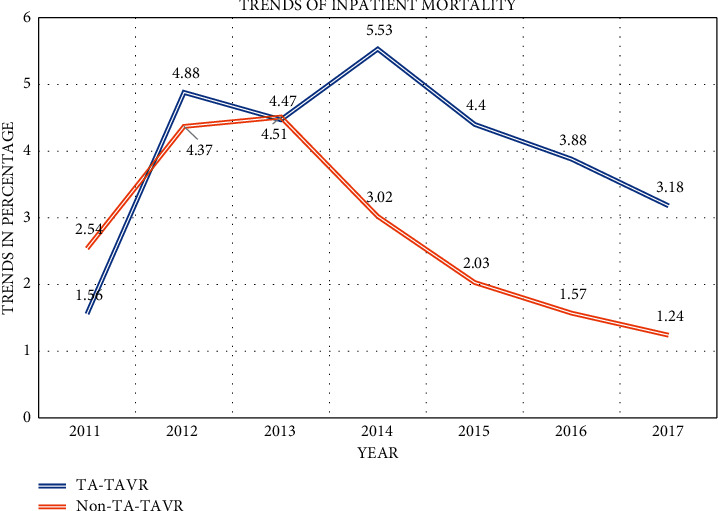
Trends of inpatient mortality from 2011–2017.

The procedural volume of TA-TAVR, after peaking in 2013 at 27.7%, declined to 1.92% in 2017 (*p* < 0.0001). Non-TA-TAVR reached a nadir in 2013 at 72.2% and increased to 98.08% in 2017. These trends are represented in Figure 1. Similarly, the trend of inpatient mortality decreased nonsignificantly in TA-TAVR group from a peak of 5.53% in 2014 to 3.18 in 2017 (*p*=0.688). The decrease of mortality trends in non-TA-TAVR was significant from a peak of 4.51% in 2013 to 1.24% in 2017 (*p*=0.0001). These trends are represented in Figure 2. The overall odds ratio (OR) of inpatient mortality for TA-TAVR after multivariable regression analysis was 1.60, with a 95% CI: 1.20–2.13, *p*=0.01 (supplementary file, Table S2). The length of stay and cost of hospitalization were also higher for TA-TAVR (9.15 ± 0.18 days vs. 5.29 ± 0.64 days, *p* < 0.0001 and $246861 ± 5417 vs. $213216 ± 2944, *p* < 0.0001, respectively).

**Figure 3:**
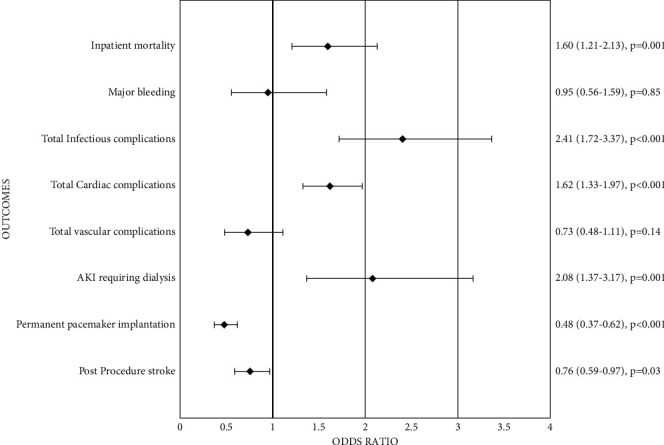
Comparison of outcomes.

In comparison of complications using multivariable regression analysis, patients undergoing TA-TAVR had higher cardiac and infectious complications after procedure, whereas the bleeding and vascular complications were comparable between the two groups. Patients undergoing TA-TAVR had significantly lower rates of postprocedure stroke or TIA and permanent pacemaker implantation as compared to patients undergoing non-TA-TAVR (Figure 3).

## 4. Discussion

The study demonstrates the declining trends of TA-TAVR over the course of 7 years when compared to the use of other access sites (predominantly TF) for TAVR. Among secondary outcomes, TA-TAVR showed a nonsignificant decline in in-hospital mortality, whereas in non-TA-TAVR, it decreased significantly. The length of stay and cost were higher in TA-TAVR and many complications also showed higher trends when using TA access for TAVR. After the PARTNER trial was published in 2010, our study showed an initial increase in the number of TAVRs done through TA access until 2013, following which the numbers reduced drastically. By the time the PARTNER II trial was published in 2016, the numbers for TA-TAVR were reduced to 3.2% of all TAVRs and declined to a mere 1.9% in 2017. Several reasons can be hypothesized for this fall in a number of volumes and will be discussed here in detail.

One of the most important reasons behind this decline is the in-hospital mortality trends, which never fell below 3% for TA-TAVR. Our study showed that though over the course of 7 years, the in-hospital mortality trends showed some decline (except from year 2011 to 2012), this decrease was not statistically significant (5.53% in 2014 vs. 3.1% in 2017, *p* = 0.69), whereas for other access sites (especially TF), the trends fell significantly from a peak of 4.3% in 2012 to mere 1.2% in 2017 (*p* < 0.0001). The peak use of TA-TAVR in 2013 was immediately followed by a peak in its mortality rates in 2014, and physicians performing TAVR were quickly able to recognize it, following which the use of TA access steadily declined (Figures 1 and 2). The overall in-hospital mortality over 7 years was significantly higher in TA-TAVR when compared to other access sites (4.47% vs. 2.03%, *p* < 0.0001). Even though TA access has been used for higher-risk populations such as patients with chronic lung disease, atrial fibrillation, or peripheral vascular disease (Table 2), our study showed that the risk of in-hospital mortality remained elevated even after adjusting for baseline characteristics in the multivariate regression analysis (OR 1.60, 95% CI: 1.21–2.13, *p* = 0.001). These results are similar to other studies where TA-TAVR has been shown to have higher mortality rates as compared to TF TAVRs [8,9]. An analysis of the French TAVR registry by Gilard et al. showed a lower mortality risk at 30 days and 6 months for TF-TAVR when compared to TA-TAVR (HR 0.68, 95% CI 0.55–0.83, *p* < 0.001) [11]. Similar results were also seen in PARTNER-I patients where the mortality was higher in TA-TAVR patients 6 months after procedure (19% vs. 12%, *p* = 0.01) [8].

Higher complication rates are another reason for the decline in the volume of TA-TAVR. Our study demonstrated higher rates of cardiac (OR: 1.62, 95% CI: 1.33–1.97, *p* < 0.001) or infectious complications (OR: 2.41, 95% CI: 1.72–3.37, *p* < 0.001) and AKI requiring dialysis (OR: 2.08, 95% CI: 1.37–3.17, *p*=0.001) in TA-TAVR group. Not only this, but the length of stay (9.15 ± 0.18 days vs. 5.29 ± 0.64 days, *p* < 0.0001) also remained higher in the TA-TAVR group and increased cost ($246,861 ± 5417 vs. $213,216 ± 2944, *p* < 0.0001), which could be the result of the higher number of complications. Similar results in increased length of stay were shown by Blackstone et al. [8] for TA-TAVR (8 vs. 5 days, *p* < 0.0001). Studies by Biancari et al. [9] and Schymik et al. [12] also showed higher rates of acute kidney injury (44.4% vs. 21.9%, *p* < 0.0001, and OR 2.81, 95% CI: 1.93–4.09, respectively) in TA-TAVR.

The third and most important reason which led to the further demise of the TA approach was improvisation and growing familiarity with other forms of access. As the newer generation device size became smaller, the sheath size became smaller, thus decreasing complications for TF-TAVR and making it more attractive and compatible [13]. The advent of new sites such as trans-axillary access, which had a lower rate of complications than TA started pushing physicians away from the use of apical access. This was validated in the study by Dahle et al. where the rate of use of trans-axillary access increased from 20.2% in 2015 to 49% in 2017 [14]. The introduction of intravascular lithotripsy-mediated facilitation of femoral access for TAVR now provides another option for physicians to use TF access which earlier was limited due to peripheral arterial disease [15–17]. Thus, a combination of all these factors provides a deep insight into understanding the declining trend of TA-TAVR provided by this study.

## 5. Conclusion

This study demonstrated the decline in utilization of trans-apical access for transcatheter aortic valve replacement along with higher inpatient mortality, increased length of stay, and higher costs in these patients. This combined with improvisation and availability of alternative access sites have contributed towards the near-end of an era of TA-TAVR.

## 6. Limitations

First, the study is retrospective and the comparison between the two groups was not done in a randomized way. Second, the study is based on administrative data and is highly dependent on coding, and inconsistencies during the process of data entry cannot be ruled out. Third, clinical predictors and severity of outcomes cannot be assessed as NIS does not provide information on these parameters. Fourth, ICD codes are not available for non-TA or non-TF sites. Many physicians code alternative access site procedures in the TF-TAVR ICD codes, thus an accurate measure of individual site volumes is not available. Lastly, there is an inherent selection bias as the patients who were chosen for TA-TAVR may not be candidates for non-TA-TAVR and this may impact the outcomes data despite multivariable analysis. Despite these limitations, this study is one of the largest studies providing data on the outcomes of TA-TAVR against non-TA-TAVR.

## Figures and Tables

**Table 1:** Demographics and hospital characteristics in patients undergoing TAVR through transapical vs. nontransapical approaches.

	Non-TA-TAVR	TA-TAVR	*p* value
Total number of procedures	119,663	10,158	

Mean age (years)	80.65 ± 0.06	79.71 ± 0.23	0.0001

*Gender* (%)			
Female	45.53	50	0.0002

Race (%)			
White	87.3	87.19	0.25
Black	3.99	3.35	
Hispanic	4.22	4.99	

*Primary expected payer* (%)			0.13
Medicare	90.72	88.95	
Medicaid	1.05	1.25	
Private insurance	6.43	7.66	
Self-pay	0.44	0.75	
No charge	0.02	0.05	
Other	1.34	1.34	

*Region of hospital* (%)			0.25
Northeast	24.18	26.44	
Midwest	22.98	19.27	
South	33.81	34.55	
West	19.03	19.74	

*Hospital bed size* (%)			0.84
Small	5.51	6.02	
Medium	18.11	17.58	
Large	76.39	76.4	

*Median household income national quartile for patient ZIP code* (%)			0.99
0–25^th^ percentile	21.16	21.23	
26^th^ to 50^th^ percentile	25.47	25.51	
51^st^ to 75^th^ percentile	26.2	26.25	
76^th^ to 100^th^ percentile	27.18	27	

**Table 2:** Comorbidities of patients undergoing TAVR through transapical vs. nontransapical approaches.

	Non-TA-TAVR	TA-TAVR	*p* value
Hypertension	52.19	74.15	0.0001
Type 2 DM	37.38	34.57	0.0109
Chronic ischemic heart disease	40.23	62.39	0.0001
Acute myocardial infarction	1.82	1.79	0.92
Dyslipidemia	67.48	66.4	0.37
Peripheral vascular disease	20.03	35.32	0.0001
Obesity	14.57	11.19	0.0001
Chronic kidney disease	35.16	33.13	0.12
ESRD	3.56	3.88	0.48
Atrial fibrillation	31.59	44.62	0.0001
Chronic liver disease	1.53	1.24	0.32
Chronic lung disease	32.87	42.79	0.0001
Smoking status	34.77	33.52	0.31
Alcohol consumption	0.75	0.5	0.19

## Data Availability

The data used to support the findings of this study are publicly available on National Inpatient Sample database.

## Supplementary Materials

Supplementary Materials Table S1: a tabulated version of the ICD codes used to define each complication. The definitions of each complication are also added to the table. ICD: International Classification of Diseases, GI: gastro-intestinal, GU: genito-urinary, TIA: transient ischemic attack, AKI: acute kidney injury. Table S2: multivariable analysis of inpatient mortality of patients undergoing TAVR. TAVR: transcatheter aortic valve replacement, DM: diabetes mellitus, MI: myocardial infarction, CABG: coronary artery bypass graft, PCI: percutaneous coronary intervention, CKD: chronic kidney disease, ESRD: end stage renal disease, Afib: atrial fibrillation, AKI: acute kidney injury.
