# Supplementary material for: Declining Trend of Transapical Access for Transcatheter Aortic Valve Replacement in Patients with Aortic Stenosis
Source: J Interv Cardiol. 2022 Sep 19;2022:5688026. doi: 10.1155/2022/5688026 (PMC9553752; doi:10.1155/2022/5688026)
Supplement: Supplementary Materials — Table S1: a tabulated version of the ICD codes used to define each complication. The definitions of each complication are also added to the table. ICD: International Classification of Diseases, GI: gastro-intestinal, GU: genito-urinary, TIA: transient ischemic attack, AKI: acute kidney injury. Table S2: multivariable analysis of inpatient mortality of patients undergoing TAVR. TAVR: transcatheter aortic valve replacement, DM: diabetes mellitus, MI: myocardial infarction, CABG: coronary artery bypass graft, PCI: percutaneous coronary intervention, CKD: chronic kidney disease, ESRD: end stage renal disease, Afib: atrial fibrillation, AKI: acute kidney injury. [file 5688026.f1.docx]

**Supplementary files:**

**SF Table 1:** Shows a tabulated version of the ICD codes used to define each complication. The definitions of each complication are also added to the table. ICD: International Classification of Diseases, GI: Gastro-intestinal, GU: Genito-urinary, TIA: Transient ischemic attack, AKI: Acute Kidney Injury

|  | ICD-9 Codes | ICD-10 Codes |
| --- | --- | --- |
| Total major bleeding  Defined as composite of GI bleeding, GU bleeding, intracranial bleeding or other bleeding (hemoptysis, epistaxis, hematoma unspecified hemorrhage, etc ) requiring blood transfusion | GI Bleed (530.21,456.0,530.7, 530.82 , 578.0, 578.1 , 578.9 , 456.20 ,531.00 ,531.01, 531.20, 531.21 ,531.40, 531.41 ,531.60, 531.61 ,532.00 ,532.01 ,532.21 ,532.40 ,532.41 ,532.60,532.61 ,533.00,533.01 ,533.20 ,533.21 ,533.40 ,533.41 ,533.60 ,533.61 ,534.00 ,534.41 ,534.60 ,534.61 ,535.01 ,535.11 ,535.21 ,535.31 ,535.41 ,535.51 ,535.61 ,535.71 ,537.83 ,562.02 ,562.03 ,562.12 ,562.13 ,569.3 ,569.85 ,537.84 ,569.86), GU Bleed (596.7 ,599.70 ,599.71) ,Post-op hematoma/hemorrhage (998.11, 998..12), Hemoptysis (786.30), Epistaxis (784.7), Unspecified hemorrhage (459.0), Intracranial hemorrhage (430, 432.0,432.1,432.9), Blood transfusion (99.00, 90.02,99.03,90.04) | GI Bleed (K66.1, K25.0, K25.2, K26.0, K26.2, K27.0, K27.2, K28.0, K28.2, K29.01, K29.21, K29.61, K29.71, K29.81, K29.91, K31.811, K31.82, K55.21, K57.01, K57.11, K57.13, K57.21, K57.31, K57.33, K57.41, K57.51, K57.53, K57.81, K57.91, K57.93, K62.5), GU Bleed (N32.89, R31.9, R31.0), Post-op hematoma/ hemorrhage (I97.410, I97.411, I97.418, I97.42, I97.610, I97.611, I97.618, I97.620), Hemoptysis (R04.2, R04.9), Epistaxis (R04.0), Unspecified hemorrhage (R58), Intracranial hemorrhage (I60.00, I60.01, I60.02, I60.10, I60.11, I60.12, I60.30, I60.31, I60.32, I60.4, I60.50, I60.51, I60.52, I60.6, I60.7, I60.8, I60.9, I61.0, I61.1, I61.2, I61.3, I61.4, I61.5, I61.6, I61.8, I61.9, I62.00, I62.01, I62.02, I62.03, I62.1, I62.9), Blood transfusion (30230H0, 30230H1, 30230K0, 30230K1, 30230L0, 30230L1, 30230M0, 30230M1, 30230N0, 30230N1, 30230P0, 30230P1, 30230R0, 30230R1, 30230T0, 30230T1, 30233H0, 30233H1, 30233K0, 30233K1, 30233L0, 30233L1, 30233M0, 30233M1, 30233N0, 30233N1, 30233P0, 30233P1, 30233R0, 30233R1, 30233T0, 30233T1, 30240H0, 30240H1, 30240K0, 30240K1, 30240L0, 30240L1, 30240M0, 30240M1, 30240N0, 30240N1, 30240T0, 30240T1, 30243H0, 30243H1, 30243K0, 30243K1, 30243L0, 30243L1, 30243M0, 30243M1, 30243N0, 30243N1, 30243P0, 30243P1, 30243R0, 30243R1, 30243T0, 30243T1) |
| Sepsis (Infectious complications) | 038.0, 038.1, 038.10, 038.11,038.12,038.19,038.2, 038.3, 038.40, 038.41, 038.42, 038.43, 038.44, 038.49, 038.8, 038.9, 995.91, 995.92, 785.52 | T80.211A, A40.0, A40.3, A40.8, A40.9, A41.4, A41.50, A41.59, A41.51, A41.52, A41.53, A41.81, A41.89, A41.9, R65.10, R65.11, R65.20, R65.21, T81.10XA, T81.12XA, T81.40XA, T81.42XA, T81.43XA, T81.44, T81.49XA |
| Cardiac complications  Composite of Iatrogenic Cardiac Complications, heart block requiring pacemaker, hemopericardium, cardiac tamponade | Iatrogenic cardiac complications (997.1), Complete heart block (426.0) requiring pacemaker (37.81,37.73, 37.82, 37.71, 37.83, 37.72, 37.78) hemopericardium (423.0), cardiac tamponade (423.3) | Iatrogenic cardiac complications (I97.710, I97.790, I97.788,I97.789), Complete heart block(I44.2), requiring Pacemaker (0JH605Z, 0JH635Z, 0JH805Z, 0JH835Z, 02H64JZ, 02H63JZ, 02HK3JZ, 02HK4JZ, 0JH606Z, 0JH636Z, 0JH806Z, 0JH836Z), hemopericardium (I31.2, S26.00XA, S26.01XA, S26.020A, S26.021A, S26.022A, S26.09XA), Cardiac tamponade (I31.4) |
| Total vascular complication  (Includes injury to blood vessels, accidental puncture, other vascular complications) | 900.00, 900.01, , 900.02, 900.03, 900.1, 900.81, 900.82, 900.89, 900.9, 901.0, 901.1, 901.2, 901.3, 901.40, 901.41, 901.42, 901.81, 901.82, 901.83, 901.89, 901.9, 902.0, 902.10, 902.11, 902.19, 902.20, 902.21, 902.22, 902.23, 902.24, 902.25, 902.26, 902.27, 902.29, 902.31, 902.32, 902.33, 902.34, 902.39, 902.40, 902.41, 902.42, 902.49, 902.50, 902.51, 902.52, 902.53, 902.54, 902.55, 902.56, 902.59, 902.81, 902.82, 902.87, 902.89, 902.9 , 903.00, 903.01, 903.02, 903.1, 903.2, 903.3, 903.4, 903.5, 903.8, 903.9, 904.0, 904.1, 904.2, 904.3, 904.41, 904.42, 904.50, 904.51, 904.52, 904.53, 904.54, 904.6, 904.7, 904.8, 904.9, 998.2, E870.0, E871.0, E872.0, 999.2 | S15.001A, S15.002A, S15.009A, S15.011A, S15.012A, S15.019A, S15.021A, S15.022A, S15.029A, S15.091A, S15.099A, S15.8XXA, S15.9XXA, S35.00XA, S35.01XA, S35.02XA, S35.09XA, S75.001A, S75.002A, S75.009A, S75.011A, S75.012A, S75.019A, S75.021A, S75.022A, S75.029A, S75.091A, S75.092A, S75.099A, I97.51, I97.52, T81.719A |
| Post-op stroke or TIA | 997.02, 435.0, 435.1 , 435.2, 435.8, 435.9 ,V125.4 | I97.810, I97.811, I97.820, I97.821 |
| AKI requiring dialysis | AKI (584.6, 584.7, 584.8, 584.9,586.0, 997.5) Hemodiyalsis (39.95) | AKI (N17.0, N17.1, N17.2, N17.8, N17.9) Hemodialysis (5A1D00Z, 5A1D60Z) |
| Permanent pacemaker | 37.81,37.73, 37.82, 37.71, 37.83, 37.72, 37.78 | 0JH605Z, 0JH635Z, 0JH805Z, 0JH835Z, 02H64JZ, 02H63JZ, 02HK3JZ, 02HK4JZ, 0JH606Z, 0JH636Z, 0JH806Z, 0JH836Z |

**SF Table 2:** Multivariable analysis of inpatient mortality of patients undergoing TAVR. TAVR: Transcatheter Aortic Valve Replacement, DM: Diabetes Mellitus, MI: Myocardial Infarction, CABG: Coronary Artery Bypass Graft, PCI: Percutaneous Coronary Intervention, CKD: Chronic Kidney Disease, ESRD: End Stage Renal Disease, Afib: Atrial Fibrillation, AKI: Acute Kidney Injury

|  | OR (95% CI) | t | p>\|t\| |
| --- | --- | --- | --- |
| Transapical approach | 1.60 (1.20-2.13) | 3.25 | 0.001 |
| Age | 1.02 (1-1.03) | 2.57 | 0.010 |
| Female | 1.12 (0.91-1.38) | 1.16 | 0.246 |
| Hypertension | 0.79 (0.64-0.97) | -2.20 | 0.028 |
| Type 2 DM | 0.76 (0.61-0.93) | -2.57 | 0.010 |
| Chronic ischemic heart disease | 1.63 (1.33-2) | 4.70 | <0.001 |
| Acute MI | 2.64 (1.67-4.18) | 4.16 | 0.001 |
| Dyslipidemia | 0.65 (0.53-0.79) | -4.19 | 0.001 |
| H/o CABG | 0.84 (0.64-1.11) | -1.20 | 0.231 |
| H/o PCI | 0.73 (0.51-1.05) | -1.66 | 0.097 |
| Peripheral vascular disease | 0.98 (0.78-1.22) | -0.15 | 0.88 |
| Obesity | 0.80 (0.57-1.12) | -1.28 | 0.20 |
| CKD | 1.23 (1-1.51) | 1.98 | 0.04 |
| ESRD | 1.03 (0.61-1.72) | 0.12 | 0.90 |
| A.Fib | 1.04 (0.86-1.27) | 0.47 | 0.63 |
| Chronic liver disease | 1.70 (0.94-3.09) | 1.76 | 0.07 |
| Chronic lung disease | 1.40 (1.15-1.71) | 3.35 | 0.001 |
| Smoking | 0.63 (0.50-0.79) | -3.96 | 0.001 |
| Alcohol | 0.49 (0.11-2.17) | -0.92 | 0.356 |
| Major bleeding | 2.65 (1.33-5.27) | 2.80 | 0.005 |
| Total infectious complication | 13.04 (9.31-18.27) | 14.93 | 0.001 |
| Cardiac complication | 4.32 (3.25-5.73) | 10.15 | 0.001 |
| Thromboembolic complication | 1.35 (0.95-1.93) | 1.70 | 0.089 |
| Total vascular complication | 6.07 (4.12-8.96) | 9.11 | 0.001 |
| Iatrogenic respiratory complication | 1.71 (1.01-2.88) | 2.02 | 0.044 |
| Post procedure stroke | 1.08 (0.67-1.76) | 0.33 | 0.744 |
| AKI injury requiring dialysis | 6.58 (3.88-11.17) | 6.99 | 0.001 |
| Permanent pacemaker | 0.29 (0.18-0.44) | -5.56 | 0.001 |
